# Relationship of different platelet response criteria and patient outcomes in a romiplostim myelodysplastic syndromes trial

**DOI:** 10.1038/leu.2014.253

**Published:** 2014-09-23

**Authors:** U Platzbecker, M A Sekeres, H Kantarjian, A Giagounidis, G J Mufti, C Jia, A S Yang, P Fenaux

**Affiliations:** 1 University Hospital Carl Gustav Carus Dresden, Medizinische Klinik und Poliklinik I, Dresden, Germany; 2 Leukemia Program, Cleveland Clinic Taussig Cancer Institute, Cleveland, OH, USA; 3 Department of Leukemia, The University of Texas MD Anderson Cancer Center, Houston, TX, USA; 4 Clinic for Oncology, Hematology and Palliative Medicine, Marien Hospital Düsseldorf, Düsseldorf, Germany; 5 King's College London, London, UK; 6 Amgen Inc., South San Francisco, CA, USA; 7 Amgen Inc., Thousand Oaks, CA, USA; 8 Service d'hématologie clinique, Hopital Avicenne Universite Paris XIII, Bobigny, France

Thrombocytopenia in lower-risk myelodysplastic syndrome (MDS) contributes to an increased risk of bleeding and is associated with shortened survival.^1, 2, 3^ Empiric platelet response criteria have been used in MDS clinical trials mostly with disease-modifying drugs, often as surrogates for clinical outcomes (Table 1a). The value of these criteria has not been rigorously evaluated, most importantly not in trials of agents specifically targeting platelet production. Romiplostim is currently approved in the United States for the treatment of thrombocytopenia in patients with chronic immune thrombocytopenia (ITP) who have had an insufficient response to corticosteroids, immunoglobulins or splenectomy, and is marketed under the name Nplate. Results of trials of romiplostim in MDS suggest that romiplostim treatment improves platelet counts as monotherapy and when combined with azacitidine, decitabine or lenalidomide.^4, 5, 6, 7^

Data from a 58-week placebo-controlled trial of romiplostim in patients with lower-risk MDS and thrombocytopenia^7^ were used to evaluate how the platelet response measures associate with clinical outcomes. This trial was discontinued early owing to concerns that the potential small benefit of bleeding reduction did not outweigh the potential risk for diagnosis of acute myeloid leukemia (AML) as defined by pathology or initiation of AML-type treatment; the final analysis of the 58-week data set showed comparable AML rates in both arms.^7^ We describe here to what extent the currently clinically available platelet response criteria were applicable in this study and whether they were associated with platelet transfusions, bleeding and overall survival.

Patients with International Prognostic Scoring System (IPSS) low/int-1 risk MDS (*N*=250) were randomized 1:2 to 26 weeks of weekly placebo (*N*=83) or romiplostim (*N*=167), with dose adjusted for platelet count, followed by a 4-week washout period and bone marrow biopsy, another 24 weeks as randomized (extended treatment period), and a second 4-week washout period and bone marrow biopsy (ClinicalTrials.gov NCT00614523).^7^ The 4-week washouts occurred before bone marrow biopsies so that the study drug would not affect results, as romiplostim can be associated with transient blast cell count elevations. Patients then entered long-term follow-up.

Eligible patients were receiving supportive care only (that is, not disease-modifying therapy), with platelet counts (1) ⩽20 × 10^9^/l or (2) ⩽50 × 10^9^/l and a history of bleeding. Patients were stratified by baseline IPSS status (low, int-1) and platelet count (<, ⩾20 × 10^9^/l). Platelet response was assessed using various platelet response criteria (Table 1a). Clinically significant bleeding events (CSBEs) were defined as grade ⩾2 on the modified World Health Organization (WHO) bleeding scale.^8,9^

All analyses were performed *post hoc*. The Fisher's exact test was used to test the association of romiplostim and platelet response. The Mantel–Haenszel method was used to calculate a pooled odds ratio of romiplostim and placebo across the randomization stratification factors of baseline platelet count and IPSS. A Cox regression model including prognostic factors and platelet response as covariates was used to predict overall survival in romiplostim-treated patients. Poisson regression models, including baseline platelet count, IPSS status and platelet response as covariates, were used to predict bleeding events and platelet transfusions in romiplostim-treated patients. As this was an analysis based on response, a landmark sensitivity analysis was performed using platelet counts from the first 26 weeks to determine platelet response, excluding patients who discontinued the trial within 26 weeks, and analyzing outcomes that occurred after 26 weeks; survival curves by response status were plotted per landmark at week 26. CSBEs were too infrequent for the landmark sensitivity analysis to be meaningful. Overall survival was recorded up to the last observation in long-term follow-up, and bleeding events and platelet transfusions were measured in the extended treatment period only.

The six platelet response measures (Table 1a) were used to evaluate changes in platelet counts in the 58-week placebo-controlled trial in *post hoc* analyses. Patients (placebo, *N*=83; romiplostim, *N*=167) were mostly male (59.2%) and Caucasian (94.0%). Median (Q1, Q3) age was 70.0 (61.0, 77.0) years and median (Q1, Q3) baseline platelet count was 19.3 (12.5, 30.3) × 10^9^/l. Median (Q1, Q3) MDS duration was 0.44 (0.13, 1.74) years. Most patients were MDS WHO classification refractory cytopenia with multilineage dysplasia (67.6%).

Romiplostim treatment was significantly associated with platelet response by all criteria studied (Table 1b). For example, romiplostim-treated subjects were 15.6 times more likely to have hematologic improvement—platelet (HI-P) than placebo-treated subjects. All platelet response criteria also reflected whether patients required platelet transfusions, with nonresponders having more platelet transfusions than responders.

The association between platelet response criteria and clinical outcomes such as bleeding (all and CSBE) were evaluated, as in Table 1b. All response criteria showed significant association between response status and overall bleeding events, with nonresponders being more likely than responders to have bleeding events. Only HI-P, complete response as presented by the Italian MDS group,^10^ and durable response were significantly associated with less CSBE. These same measures, and International Working Group (IWG) 2000 criteria, were significantly associated with improved overall survival. Survival curves for HI-P, the platelet response measure most significantly associated with survival, are shown in Figure 1 for romiplostim-treated patients. AML rates for romiplostim-treated patients with HI-P as compared with those without HI-P were similar, 8.5% vs 8.3%, with an odds ratio (95% confidence interval) of 1.02 (0.31, 3.38).

Landmark sensitivity analyses were performed for all measures described above to determine whether this being an analysis based on response and discontinuation of patients affected trial results. For the overall survival end point, after excluding patients who discontinued in the first 26 weeks, the sample size decreased from 167 to 143. Among the 24 subjects who were excluded, 12 died. Smaller sample size and fewer events contributed to slightly larger *P*-values, although results were generally consistent with the original analyses. Differences included that complete response^10^ and IWG 2000 major response^11^ were marginally significantly associated with overall survival (*P*=0.077 and 0.053, respectively). All platelet response measures remained significantly associated with all bleeding and platelet transfusions (data not shown).

Data from this large placebo-controlled romiplostim trial indicate that platelet response criteria, developed empirically from clinical experience and trials using disease-modifying agents, are heterogeneous, result in a wide range of response rates for the same patient population and are predictive of clinical outcomes in thrombocytopenic MDS patients treated with a thrombopoietin mimetic. This is in keeping with the finding that thrombocytopenia *per se* has been associated with worse prognosis in MDS, including an increased risk of disease progression.^1, 2, 3^ For the first time, we show that platelet response to a thrombopoietin mimetic is positively associated with overall survival. Possibly, romiplostim has a beneficial effect through reducing potentially life-threatening thrombocytopenia or other as-yet-unrecognized broader effects. Thrombopoietin has previously been shown to stimulate other hematopoietic lineages.^12^ It is unclear whether the improved outcomes are associated with response to romiplostim or that, inherently, patients that respond have better outcomes. While no difference in survival was seen with romiplostim vs placebo,^7^ better selection of patients could lead to improved survival outcomes with romiplostim.

A positive association between treatment and survival is also seen for the disease-modifying therapy azacitidine in higher-risk MDS.^13^ For studies examining survival and treatment with erythropoiesis-stimulating agents (ESAs) in MDS, results have been mixed. A retrospective multivariate analysis of patients treated with ESAs with or without granulocyte colony-stimulating factors (G-CSFs) reported that survival, but not disease progression, was improved in the ESA-responsive cohort compared with an untreated IPSS/IMRAW (International MDS Risk Analysis/Workshop) cohort.^14^ Another multivariate analysis, comparing patients treated with ESAs plus G-CSFs with a control cohort of untreated MDS patients, found better survival with ESA treatment, particularly for those requiring fewer than two red blood cell units transfused per month.^15^ However, a small randomized phase 3 Eastern Cooperative Oncology Group (ECOG) study of patients receiving supportive care alone or supportive care plus ESAs with or without G-CSFs found no difference in survival, and that survival was increased for those who responded to ESA treatment.^16^ Whether ESA treatment improves survival in MDS may become clearer as ongoing studies report results.

In summary, these data indicate that platelet response criteria, specifically those that incorporate durable response, such as IWG 2006, correlate with overall survival and have the potential to be used as interim markers for clinically significant outcomes. However, a limitation of this data set is that the study drug treatment was ended early owing to concerns regarding transient increases in peripheral blast cell counts with romiplostim that put patients at risk for the diagnosis of and treatment for AML. Therefore, the data set was incomplete, and it is possible that different results regarding the association of platelet response measures and clinical outcomes would have been obtained with a fuller data set. Evaluation is needed of these associations in either past MDS clinical trials of interventions to raise platelets or future ones to confirm that these findings occur in the broader context.

## Figures and Tables

**Figure 1:**
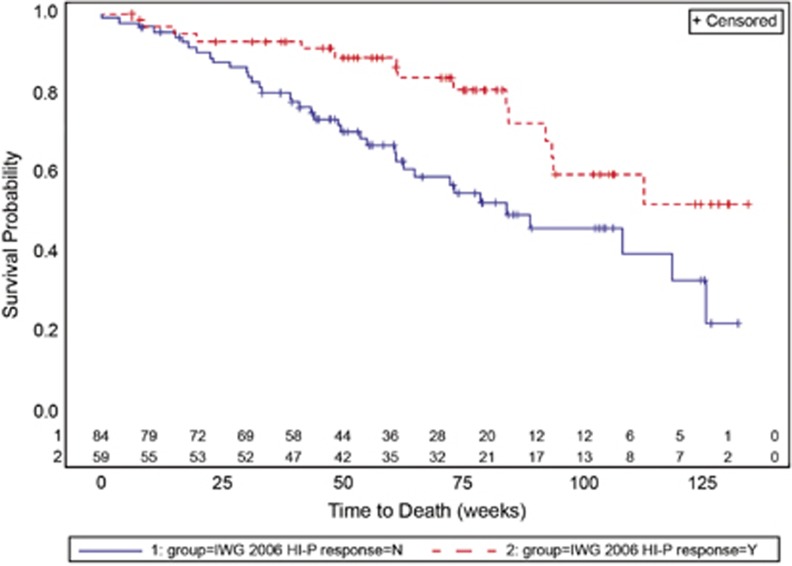
Survival curves. Shown are the survival probability for those who had achieved an IWG 2006 HI-P response (red dashed line) and those who did not (solid blue line), all in romiplostim-treated patients. Number of patients at risk is shown on the graph immediately above the *x* axis. *P*=0.0070 by log-rank test.

**Table 1a:** Platelet response criteria evaluated

*Platelet response criteria*	*Duration*	*Definition*
IWG 2006 HI-P^17^	8 weeks	For patients with baseline platelets <100 × 10^9^/l: Baseline platelets >20 × 10^9^/l, absolute increase of ⩾30 × 10^9^/l Baseline platelets <20 × 10^9^/l, increase to >20 × 10^9^/l and by ⩾100%
IWG 2000 Major^11^	8 weeks^a^	For patients with baseline platelets <100 × 10^9^/l: An absolute increase of ⩾30 × 10^9^/l For platelet transfusion-dependent patients, stabilization of platelet counts and platelet transfusion independence
IWG 2000 Minor^11^	8 weeks^a^	For patients with baseline platelets <100 × 10^9^/l: A ⩾50% increase in platelet count with an absolute increase >10 × 10^9^/l but <30 × 10^9^/l
		
*Italian MDS group*^10^		
Complete (same as IWG AML 2003^18^)	None	Platelet count >100 × 10^9^/l and no bleeding
Any	None	Baseline platelets >20 × 10^9^/l, no bleeding and absolute increase of ⩾30 × 10^9^/l Baseline platelets <20 × 10^9^/l, increase to >20 × 10^9^/l and by at least 100%
Durable	4 weeks	Continuous platelet response
CALGB^19^	None	⩾50% restitution of the initial deficit (to 140 × 10^9^/l)
ITP^20^	None	Platelet count>50 × 10^9^/l

Abbreviations: AML, acute myeloid leukemia; CALGB, Cancer and Leukemia Group B; HI-P, hematologic improvement—platelet; ITP, immune thrombocytopenia; IWG, International Working Group; MDS, myelodysplastic syndrome.

a As platelets were measured every week in this trial, for IWG 2000 response measures, an 8-week duration was used for these analyses, rather than 2 months.

**Table 1b:** Association of platelet response rates and transfusion needs with various platelet response criteria

*Platelet response criteria*	*Response rate*				*Platelet transfuse RR*^a^ *(95% CI)*	*Bleeding*^b^		*OS*^c^
	*Placebo* N*=83,* n *(%)*	*Romiplostim* N*=167,* n *(%)*	P*-value*	*OR (95% CI)*^b^		*All RR (95% CI)*	*CSBE RR (95% CI)*	*HR (95% CI)*
IWG 2006 HI-P	3 (3.6)	61 (36.5)	<0.001	15.6* (4.7–51.8)	9.8* (7.8–12.3)	2.3* (2.1–2.6)	1.4* (1.0–2.0)	2.6* (1.4–4.7)
IWG 2000 Major or Minor	2 (2.4)	56 (33.5)	<0.001	21.2* (5.0–90.3)	13.0* (9.8–17.2)	2.5* (2.2–2.8)	1.2 (0.8–1.6)	2.2* (1.2–4.1)
								
*Italian MDS group*								
Complete (IWG AML 2003)	4 (4.8)	63 (37.7)	<0.001	15.4* (5.0–47.2)	5.8* (4.7–7.2)	2.6* (2.3–2.9)	1.8* (1.2–2.7)	1.9* (1.0–3.4)
Any	26 (31.3)	115 (68.9)	<0.001	5.1* (2.8–9.1)	2.6* (2.3–2.9)	2.1* (1.9–2.3)	1.2 (0.9–1.7)	1.5 (0.9–2.6)
Durable	4 (4.8)	82 (49.1)	<0.001	19.7* (6.9–56.5)	9.5* (7.8–11.4)	2.5* (2.3–2.7)	1.8* (1.3–2.4)	2.2* (1.2–3.8)
CALGB	6 (7.2)	75 (44.9)	<0.001	12.5* (4.9–31.5)	1.8* (1.6–2.0)	2.8* (2.5–3.1)	0.8 (0.6–1.1)	1.6 (0.9–2.9)
ITP	17 (20.5)	95 (56.9)	<0.001	6.3* (3.2–12.4)	2.8* (2.4–3.2)	2.1* (1.9–2.3)	0.9 (0.7–1.2)	1.6 (0.9–2.9)

Abbreviations: AML, acute myeloid leukemia; CALGB, Cancer and Leukemia Group B; CI, confidence interval; CSBE, clinically significant bleeding event; HI-P, hematologic improvement—platelet; HR, hazard ratio; IPSS, International Prognostic Scoring System; ITP, immune thrombocytopenia; IWG, International Working Group; MDS, myelodysplastic syndrome; OR, odds ratio; OS, overall survival; RR, rate ratio.

a Romiplostim-treated patients only, responders vs nonresponders; adjusted for baseline platelet count and IPSS status.

b Romiplostim vs placebo; adjusted for baseline platelet count and IPSS status.

c Romiplostim-treated patients, nonresponders vs responders; adjusted for baseline age (65 years or older) and IPSS status. Statistically significant OR and RR are bolded with asterisks.

## Notes

UP has received honoraria from Amgen, GlaxoSmithKline (GSK), Celgene and Novartis. MAS is a member of advisory boards for Amgen and Celgene, and a speaker for Celgene. HK has research grants from Amgen. AG is a member of advisory boards and a speaker for Amgen and GSK. GJM has been a member of advisory boards and speakers bureau for Amgen, Novartis, GSK and Celgene, received research funding from Celgene and has been a consultant for GSK. CJ and ASY are employees of and stockholders in Amgen. PF has received honoraria and research grants from Amgen, Celgene, Janssen Roche, GSK and Novartis.
